# First person – Stéphanie Cottier

**DOI:** 10.1242/bio.054171

**Published:** 2020-07-03

## Abstract

First Person is a series of interviews with the first authors of a selection of papers published in Biology Open, helping early-career researchers promote themselves alongside their papers. Stéphanie Cottier is first author on ‘The yeast cell wall protein Pry3 inhibits mating through highly conserved residues within the CAP domain’, published in BiO. Stéphanie is a post-doc in the lab of Roger Schneiter at the University of Fribourg, Fribourg, Switzerland, investigating using yeast model organism to gain insight into the function of the widespread CAP protein superfamily.


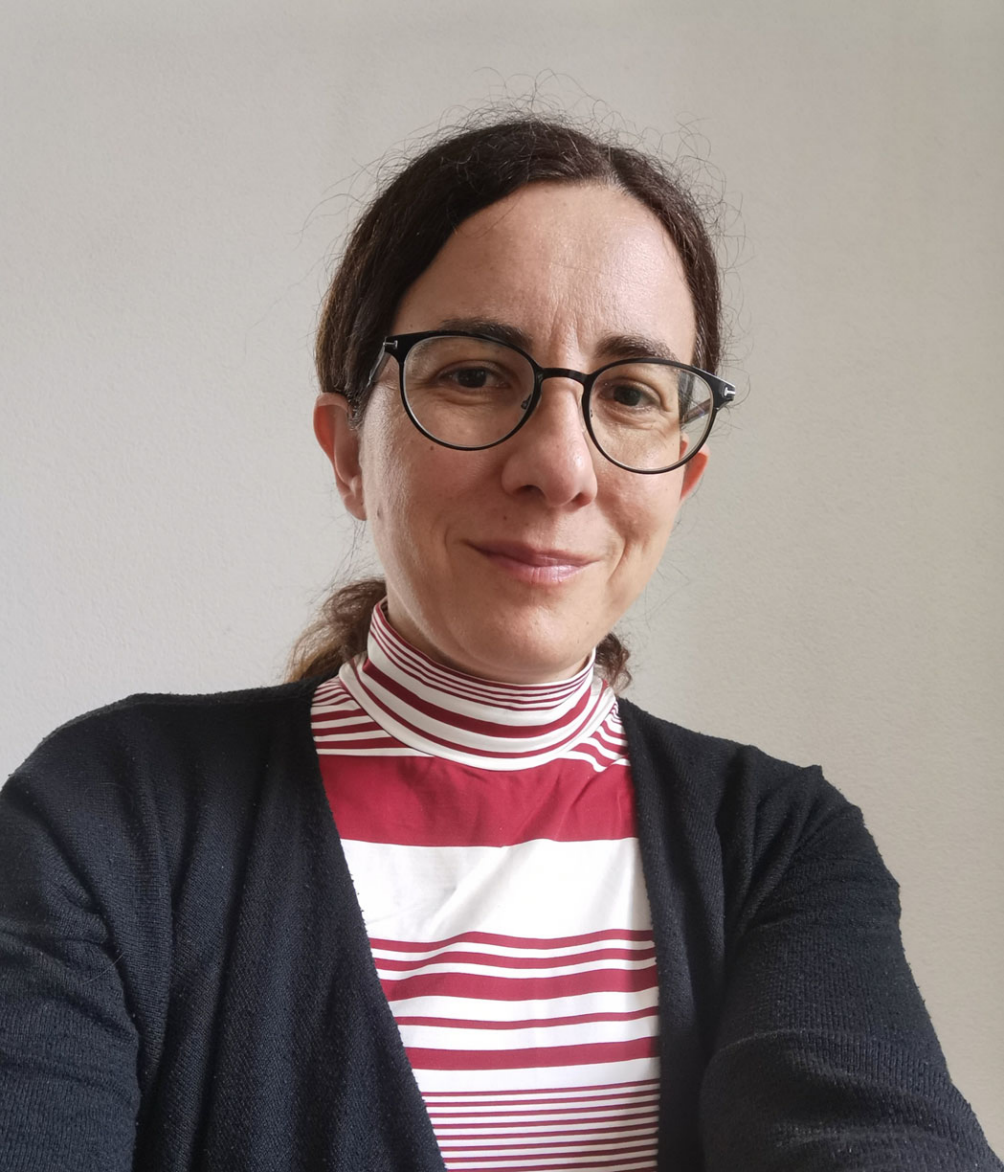


**Stéphanie Cottier**

**What is your scientific background and the general focus of your lab?**

I am a molecular and cellular biologist. I earned my PhD from the University of Heidelberg, working in Benedikt Kost's lab on polar growth in pollen tubes. Then I completed a post-doc at the Max Planck Institute for Plant Breeding Research in the laboratory of Erich Kombrink with a focus on small molecules involved in plant-pathogen interactions. Currently, I am working in the laboratory of Roger Schneiter at the University of Fribourg. For this post-doc, I have not only changed country (from Germany to Switzerland), but also the focus of my research. I am now working in a biochemistry lab studying lipid homeostasis in yeast cells. We got interested in the CAP proteins as they were identified to mediate the export of sterols in yeast. Since then we are working on the characterization of their physiological function in different organisms.

**How would you explain the main findings of your paper to non-scientific family and friends?**

I am using yeast, the same organism you are using for baking, to study the function of a family of proteins that have a common domain: the CAP domain. CAP domain proteins are not only found in yeast, but also in human, where they have been associated with fertility and cancer. It is therefore important to understand their mode of action.

My protein of interest is called Pry3. It has been shown that cells having high amounts of Pry3 have a reduced mating efficiency. We used this reduction in mating efficiency to identify the domains within Pry3, and even the single amino acids (the building blocks of proteins) within the CAP domain that are critical for this inhibition. Interestingly, these amino acids are also found in the CAP domain proteins of other organisms. We were also able to fluorescently tagged Pry3 and analysed its localization by microscopy. When Pry3 is present in high amounts, it loses its polarized localization in the cell wall, and instead spreads all over the cell surface, exerting a deleterious function on the yeast mating reaction.

**What are the potential implications of these results for your field of research?**

CAP proteins are present in many different organisms and they have been involved in a wide variety of processes including plant and mammalian immune defence, venom toxicity, sperm maturation, fertilisation, as well as in prostate and brain cancer. However, the mode of action of these conserved proteins remains largely elusive. The identification of residues in the CAP domain of Pry3 that are important for mating inhibition, and shown to be conserved in a number of CAP proteins from different organisms, gives novel cues to decipher the function of the CAP domain.

**What has surprised you the most while conducting your research?**

How complex it is to answer a tiny question. The amazing number of methods and paths nowadays science offers to do so, yet without forgetting the very basic constraints that are time and money.

**Figure:**
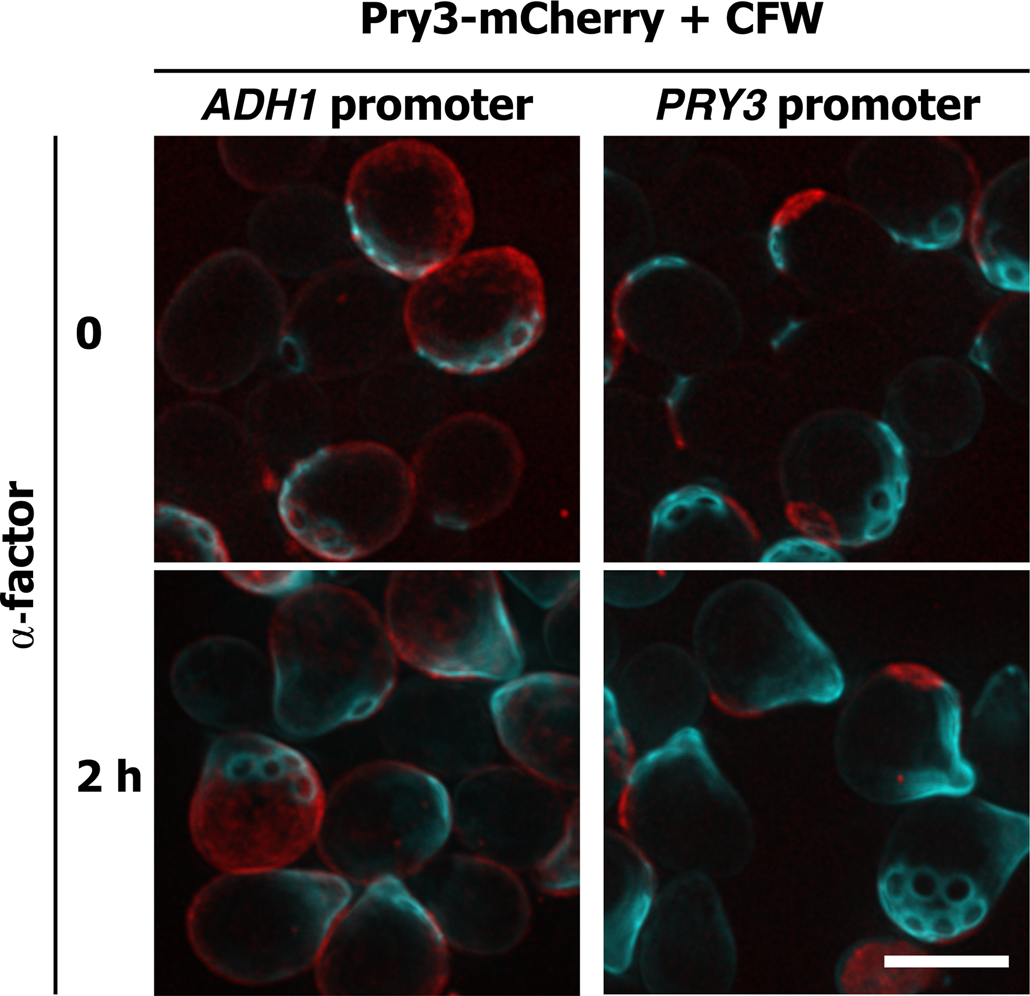
**Pry3 accumulates in the cell wall next to bud scars, and is absent from mating projection.** This highly polarized distribution is abolished when Pry3 is over-expressed. Depicted here are Pry3-mCherry and calcofluor-white (CFW) signal.

**What changes do you think could improve the professional lives of early-career scientists?**

Promoting multidisciplinary research. While collaborations are common, in my opinion they should not be limited to sharing results, but also knowledge and techniques, and give a view of different laboratory organization and philosophy.

“In research, there is always ‘the next question’!”

**What's next for you?**

We have identified residues within Pry3 that are required for mating inhibition, and we are addressing the possibility that these residues form part of a catalytic active site of the CAP domain.

In research, there is always ‘the next question’!
